# Optimising the Use of Psychotropic Coanalgesics in Frail Older Adults with Hepatorenal and/or Cardiorenal Syndromes and Neuropathic Pain: A Prototype Clinical Algorithm Developed Under the NETPHARM Project

**DOI:** 10.1192/j.eurpsy.2026.10929

**Published:** 2026-07-31

**Authors:** I. Taskova, K. Pechandova, D. Fialova

**Affiliations:** 1 Department of Social and Clinical Pharmacy, Charles University, Hradec Králové; 2 Clicnical Pharmacy Department, Psychiatric Hospital Bohnice, Prague; 3 Clicnical Pharmacy Department, Strakonice Hospital, Strakonice; 4 Department of Internal Medicine and Geriatrics, Charles University, Prague, Czech Republic

## Abstract

**Introduction:**

Frail older adults with hepatorenal and/or cardiorenal syndromes form a common yet highly challenging group regarding neuropathic pain management. The use of psychotropic drugs with coanalgesic effects is often complicated by changes in pharmacokinetics (PK), increased sensitivity to pharmacodynamic effects (PD), and drug accumulation resulting from organ dysfunctions.

**Objectives:**

We aimed to develop a training prototype of a clinical decision-making algorithm for the appropriate selection and dosing of psychotropic co-analgesics—amitriptyline, duloxetine, venlafaxine, gabapentin, pregabalin, and carbamazepine—to treat neuropathic pain in frail older adults with hepatorenal and/or cardiorenal syndromes. This work on the algorithm was conducted within the framework of the NETPHARM project.

**Methods:**

A combination of systematic and non-systematic literature searches of Medline and Web of Science databases was conducted to identify all relevant sources on PK/PD characteristics, dose recommendations, and safety profiles of selected drugs. A comparative table was created, summarising data on PK, dose adjustments in older adults, cardiovascular safety, and recommendations for use in chronic kidney disease, liver cirrhosis, and heart failure, respectively. The drugs were also evaluated regarding their inclusion in PIMs (potentially inappropriate medications) lists—Beers Criteria, STOPP/START, EU(7) (Renom-Guiteras et al. Eur J Clin Pharmacol 2015; 71:861–75; O’Mahony et al. Eur Geriatr Med 2023; 14:625–32; American Geriatrics Society. J Am Geriatr Soc 2023; 71:2052–82) —and the risk in specific frail populations (fall risk, cognitive impairment risk, orthostatic hypotension risk, etc.). Based on the comparative table, a clinical decision algorithm was developed to support rational prescribing.

**Results:**

The algorithm functions as a two-step clinical decision support system. In the first step, drug selection follows international guidelines for neuropathic pain (Attal et al. Eur J Neurol 2010; 17:1113–e88; NICE. CG173 2020; Mu et al. Can Fam Physician 2022; 68:844–50; Price et al. Neurology 2022; 98:31–43), considering specific efficacy data for each indication. The second step takes into account individual patient factors, such as frailty, the presence and severity of renal and/or hepatic impairment, heart failure, as well as the PK/PD properties of the drugs. The algorithm helps identify the most appropriate drug and dosage for each patient.

**Conclusions:**

This clinical algorithm may support safer and more personalised selection of psychotropic drugs with co-analgesic effects for managing neuropathic pain in frail older adults with hepatorenal and/or cardiorenal syndromes.

**Disclosure of Interest:**

I. Taskova Grant / Research support from: NETPHARM project (CZ.02.01.01/00/22_008/0004607), co-funded by the European Union, K. Pechandova Grant / Research support from: NETPHARM project (CZ.02.01.01/00/22_008/0004607), co-funded by the European Union, D. Fialova Grant / Research support from: NETPHARM project (CZ.02.01.01/00/22_008/0004607), co-funded by the European Union

